# Duration of New-Onset Depressive Symptoms During Medical Residency

**DOI:** 10.1001/jamanetworkopen.2024.18082

**Published:** 2024-06-21

**Authors:** Erin Kim, Brandy R. Sinco, Joan Zhao, Yu Fang, Carrie Cunningham, Elena Frank, Srijan Sen, Amy Bohnert, Tasha M. Hughes

**Affiliations:** 1 University of Michigan Medical School, Ann Arbor; 2 Department of Surgery, University of Michigan, Ann Arbor; 3 Center for Healthcare Outcomes and Policy, Michigan Medicine, Ann Arbor; 4 Michigan Neuroscience Institute, University of Michigan, Ann Arbor; 5 Department of Surgery, Massachusetts General Hospital, Boston; 6 Institute for Technology Assessment, Massachusetts General Hospital, Boston; 7 Department of Psychiatry, University of Michigan Medical School, Ann Arbor; 8 Center for Clinical Management and Research, Ann Arbor Veterans Affairs Hospital, Ann Arbor, Michigan; 9 Department of Anesthesiology, University of Michigan, Ann Arbor

## Abstract

**Importance:**

The implications of new-onset depressive symptoms during residency, particularly for first-year physicians (ie, interns), on the long-term mental health of physicians are unknown.

**Objective:**

To examine the association between and persistence of new-onset and long-term depressive symptoms among interns.

**Design, Setting, and Participants:**

The ongoing Intern Health Study (IHS) is a prospective annual cohort study that assesses the mental health of incoming US-based resident physicians. The IHS began in 2007, and a total of 105 residency programs have been represented in this national study. Interns enrolled sequentially in annual cohorts and completed follow-up surveys to screen for depression using the 9-item Patient Health Questionnaire-9 (PHQ-9) throughout and after medical training. The data were analyzed from May 2023 to March 2024.

**Exposure:**

A positive screening result for depression, defined as an elevated PHQ-9 score of 10 or greater (indicating moderate to severe depression) at 1 or more time points during the first postgraduate year of medical training (ie, the intern year).

**Main Outcomes and Measures:**

The main outcomes assessed were mean PHQ-9 scores (continuous) and proportions of physicians with an elevated PHQ-9 score (≥10; categorical or binary) at the time of the annual follow-up survey. To account for repeated measures over time, a linear mixed model was used to analyze mean PHQ-9 scores and a generalized estimating equation (GEE) was used to analyze the binary indicator for a PHQ-9 score of 10 or greater.

**Results:**

This study included 858 physicians with a PHQ-9 score of less than 10 before the start of their internship. Their mean (SD) age was 27.4 (9.0) years, and more than half (53.0% [95% CI, 48.5%-57.5%]) were women. Over the follow-up period, mean PHQ-9 scores did not return to the baseline level assessed before the start of the internship in either group (those with a positive depression screen as interns and those without). Among interns who screened positive for depression (PHQ-9 score ≥10) during their internship, mean PHQ-9 scores were significantly higher at both 5 years (4.7 [95% CI, 4.4-5.0] vs 2.8 [95% CI, 2.5-3.0]; *P* < .001) and 10 years (5.1 [95% CI, 4.5-5.7] vs 3.5 [95% CI, 3.0-4.0]; *P* < .001) of follow-up. Furthermore, interns with an elevated PHQ-9 score (≥10) demonstrated a higher likelihood of meeting this threshold during each year of follow-up.

**Conclusions and Relevance:**

In this cohort study of IHS participants, a positive depression screening result during the intern year had long-term implications for physicians, including having persistently higher mean PHQ-9 scores and a higher likelihood of meeting this threshold again. These findings underscore the pressing need to address the mental health of physicians who experience depressive symptoms during their training and to emphasize the importance of interventions to sustain the health of physicians throughout their careers.

## Key Points

**Question:**

Do depressive symptoms with new onset during residency training persist, and are they associated with the long-term mental health of physicians?

**Findings:**

In this cohort study of 858 resident physicians, new-onset depressive symptoms identified via positive screening among first-year interns persisted after completion of training at a level more than 3-fold higher than that of their counterparts without depression.

**Meaning:**

The persistence of new-onset depressive symptoms observed among interns in this study underscores the need to support mental health among physicians as they progress through training and beyond.

## Teaser

This cohort study uses survey data from the Intern Health Study to examine the association between and persistence of new-onset and long-term depressive symptoms among first-year physicians.

## Introduction

Poor mental health among physicians is a growing professional concern and a public health crisis.^1^ Each year, 400 US physicians die by suicide, translating to 1 or more physician deaths by suicide every day.^2^ Research has demonstrated that residency training, which lasts 3 to 10 years depending on the specialty pursued, is a particularly challenging time for physician mental health.^3,4^ Residency has several characteristics implicated in the development of depression, including long work hours and inflexible schedules limiting rest and recovery.^5,6,7^ It is well-established in the general population that 1 episode of depression is associated with an increased risk of future episodes (ie, the kindling hypothesis). However, this association has not been established in the setting of residency training, which may be a transient driver of depressive symptoms secondary to a challenging work environment.

Longitudinal data evaluating mental health outcomes beyond the residency training period are lacking. Specifically, whether depressive symptoms resolve in the posttraining period is unknown, as is the potential vulnerability of a segment of the physician workforce to face future episodes of depression.^3,8,9,10^ Identification of the long-term implications of new-onset depressive symptoms during the intern year (ie, the first year of residency) is critical (1) to understand the association between the training environment and the mental health of both the training and practicing physician workforces and (2) to identify opportunities for intervention before symptoms become severe.

In this study, we examined scores on the 9-item Patient Health Questionnaire (PHQ-9) longitudinally for up to 10 years among a national sample of US physicians enrolled before the start of their internship. We aimed to quantify the persistence and severity of depressive symptoms for physicians who did and did not screen positive for depression during their first year of residency training. We hypothesized that the development of new-onset depressive symptoms as an intern was not transient but that a higher burden of depressive symptoms would persist among physicians well beyond the early years of training.

## Methods

### Study Design

The Intern Health Study (IHS) is an ongoing annual cohort study that began in 2007 to assess the mental health of incoming first-year resident physicians (interns) who are based in the US.^11^ A total of 105 residency programs have been represented in this national study. The overarching aim of the IHS is to assess the psychological, genetic, and program factors involved in the onset of depression among physicians in training. A subset of physicians recruited as interns were invited to participate in follow-up surveys of their mental health on an annual basis throughout residency training and beyond (as outlined in the Participant Recruitment section). The University of Michigan Institutional Review Board approved this cohort study. Participants provided informed consent. The study followed the Strengthening the Reporting of Observational Studies in Epidemiology (STROBE) reporting guideline.

### Participant Recruitment

#### Baseline Single-Year Survey

**Figure 1.**
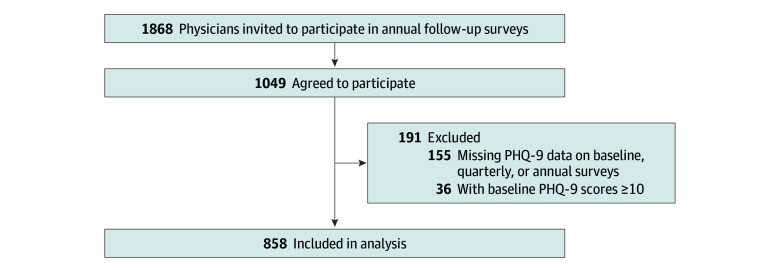
Schema for Inclusion in Annual Follow-Up Surveys PHQ-9 indicates 9-item Patient Health Questionnaire.

Beginning in 2007, cohorts of incoming interns (ie, graduating medical students) enrolled in the IHS before the start of residency training and were followed up quarterly during their first year of training. Interns were invited electronically to participate following match day, the day each year when graduating medical students learn their specialty and training location. Participants received between $50 and $125 in compensation, depending on the year they joined the study. The study questionnaires for single-year participants included a wide range of questions on mental health, training program features, and personal demographic measurements administered at baseline (3 months before the start of residency) and at the end of months 3, 6, 9, and 12 of their internship. Self-reported race and ethnicity were included in the current analysis due to prior research by our group, which has found that race and ethnicity may affect depressive symptoms among resident physicians. These categories were defined by the study investigators but self-reported by participants on the baseline survey as Asian, Black or African American, Hispanic or Latinx, White, other race or ethnicity (including American Indian or Alaska Native, Arab or Middle Eastern, Pacific Islander, other race or ethnicity, or multiple races or ethnicities), or unknown race or ethnicity. A subset of participants were then followed up for 1 assessment completed annually. Figure 1 describes the recruitment and retention of the annual follow-up survey participants.

#### Annual Follow-Up Surveys

After the initiation of the IHS in 2007, an adaptive participant recruitment strategy began in 2009 to recruit from previously enrolled interns. This recruitment strategy was designed to develop a continuous stream of annual data for each participant. At the beginning of the IHS (in 2007), the invitation was extended to all participants to participate in follow-up surveys on an annual basis. Beginning in 2014, participants from a University of Michigan biomarker substudy were invited each year, including participants from 3 previous cohorts (2010, 2011, and 2012).^12^ In 2020, recruitment was expanded to include the entire University of Michigan intern cohorts, starting with those enrolled in 2017 onward. In 2022, all surgeons from the 2009 to 2013 cohorts not already included in the annual survey were invited. All enrolled participants in the yearly follow-up study were subsequently invited to complete a survey each year until the conclusion of the study period (eTable 1 in Supplement 1). Annual surveys were not conducted during 2017 and 2021 due to funding limitations. Participants were offered $25 for each annual survey completed.

### Assessments

Upon initial enrollment in the IHS, participants completed surveys self-reporting their demographic characteristics, such as age, race and ethnicity, partnership status, program type, and specialty. The preinternship assessment included depression (measured with the PHQ-9), neuroticism (measured with the NEO Personality Inventory), history of depression among immediate family members, and personal history of medication or psychotherapy for depression.^13,14^ The PHQ-9 was used to measure depressive symptoms at baseline, at quarterly intervals across participant intern year, and annually (scores indicate the following: 0-4, none to minimal depression; 5-9, mild depression; 10-14, moderate depression; 15-19, moderately severe depression; and 20-27, severe depression).^14^ The PHQ-9 was selected due to its high sensitivity and specificity for detecting clinically meaningful depression and its comparability to clinician-administered assessments. Participants were given 1 month to complete each survey.

### Statistical Analysis

An elevated PHQ-9 score (≥10) correlates with a moderate to severe burden of depressive symptoms and is both sensitive and specific as a screening measure for moderate to severe depression (however, it is not a substitute for a clinical diagnosis of depression).^15^ Residents with an elevated PHQ-9 score (≥10) at the baseline survey, assessed 3 months before the internship, were excluded. Among the included participants with a baseline PHQ-9 score indicating none to mild depression (<10), an instance of an elevated PHQ-9 score (≥10) on at least 1 quarterly assessment during the intern year was considered a positive screening result for depression. Follow-up PHQ-9 scores on annual surveys were compared between the cohorts who did and did not meet this definition for new-onset depressive symptoms. We compared mean PHQ-9 scores and proportions of physicians who met the criteria of an elevated PHQ-9 score (≥10) between the 2 groups.

Longitudinal analysis was performed to evaluate the trajectory of mean PHQ-9 scores throughout follow-up using a linear mixed model. The difference between mean PHQ-9 scores was evaluated across 10 years of follow-up after internship completion and compared with the preinternship baseline. The percentage of residents with depression over time was analyzed using a generalized estimating equation model. The percentage of physicians with moderate to severe depressive symptoms was compared beginning 1 year after internship completion up to 10 years. Both the linear mixed model for mean PHQ-9 scores and the generalized estimating equation model for depression were adjusted for demographic characteristics, cohort year, baseline neuroticism scores, and a personal history of depression.

#### Missing Data

All longitudinal analyses were compared between the original dataset and a modified dataset with imputations across 20 variables to evaluate the effect of missing data on our results. Missing data were imputed with the fully conditional specification method. Imputation yielded more than 95% relative efficiency and did not affect our results. Therefore, original data without imputation were used for the final analysis.

#### Sample and Poststratification Weighting

Weights were computed in 2 stages, consistent with previous weighting strategies applied in this dataset.^5^ First, selection weights were calculated from propensity scores for participating in the IHS based on the distribution of race, ethnicity, and sex among potential participants at the preinternship recruitment stage. In the propensity score analysis, the outcome was designated as participation in the IHS, and the selected covariates were binary sex, race, and ethnicity (categorized as Asian, White, or other underrepresented racial or ethnic minority). Due to small samples, underrepresented minority groups were combined into a single category. The propensity score weight was calculated as 1/(propensity score).

Poststratification weights were calculated from the annual distributions of race, ethnicity, and sex from the Association of American Medical Colleges database. The overall weight was the product of the selection and poststratification weights. The final weight was the overall weight, truncated at the 95th percentile.

All analyses were performed using SAS, version 9.4 (SAS Institute Inc). The analytic code is provided in the eMethods in Supplement 1. The data were analyzed from May 2023 to March 2024.

## Results

Of the 1868 individuals invited to participate in the annual follow-up surveys, 1049 (56.2%) agreed (Figure 1). We excluded individuals who were missing PHQ-9 data on any of the annual or quarterly surveys (155 [8.3%]) or had an elevated PHQ-9 score (≥10) at baseline (36 [1.8%]), resulting in 2867 follow-up assessments of 858 individual physicians. The mean (SD) age of participants was 27.4 (9.0) years; 53.0% (95% CI, 48.5%-57.5%) were women and 47.0% (95% CI, 42.5%-51.5%) were men (Table). Participants identified as Asian (36.6% [95% CI, 32.1%-41.1%]), Black (4.4% [95% CI, 2.0%-6.7%]), Hispanic (6.1% [95% CI, 3.5%-8.7%]), White (35.3% [95% CI, 31.7%-38.9%]), or other or unknown race or ethnicity (17.6% [95% CI, 13.4%-21.8%]). The mean (SD) follow-up from the completion of the intern year was 5 (3) years. Of the 858 physicians in the final analytic sample, 302 (35.2%) reported having depressive symptoms (PHQ-9 score ≥10) during the intern year on at least 1 quarterly survey.

**Table.** Baseline Demographic Characteristics of Physicians, Weighted Data^a^

Characteristic	All physicians (N = 858)	Physicians who screened positive for depressive symptoms during intern year		*P* value^b^
		Yes (PHQ-9 ≥10) (n = 302)	No (PHQ-9 <10) (n = 556)	
Age, mean (SD), y^c^	27.4 (9.0)	27.5 (9.1)	27.3 (8.9)	.44^d^
Sex				
Female	53.0 (48.5-57.5)	59.9 (52.4-67.4)	49.1 (43.5-54.7)	.03^e^
Male	47.0 (42.5-51.5)	40.1 (32.6-47.6)	50.9 (45.3-56.5)	
Race and ethnicity				
Asian	36.6 (32.1-41.1)	36.9 (29.2-44.6)	36.4 (30.8-42.0)	.57^e^
Black or African American	4.4 (2.0-6.7)	6.2 (1.4-10.9)	3.4 (0.9-5.9)	
Hispanic or Latinx	6.1 (3.5-8.7)	4.0 (0.3-7.7)	7.3 (3.8-10.8)	
White	35.3 (31.7-38.9)	35.0 (28.9-41.2)	35.4 (30.9-40.0)	
Other or unknown^f^	17.6 (13.4-21.8)	17.9 (10.9-24.9)	17.4 (12.3-22.6)	
Married or partnered^g^	32.0 (27.8-36.2)	30.0 (22.9-37.0)	33.1 (27.9-38.4)	.48^e^
Specialty				
Surgery	51.5 (47.0-55.9)	53.1 (45.6-60.5)	50.6 (45.0-56.1)	.57^e^
Medical or pediatrics	35.7 (31.9-39.5)	36.0 (29.5-42.4)	35.5 (30.8-40.2)	
Emergency medicine	4.3 (3.0-5.6)	3.6 (1.7-5.5)	4.7 (3.0-6.4)	
Psychiatry	3.5 (2.3-4.6)	3.8 (1.6-6.0)	3.3 (2.0-4.6)	
Unknown	5.1 (3.7-6.5)	3.6 (1.9-5.3)	5.9 (4.0-7.9)	
Baseline PHQ-9 score, mean (SD)	2.1 (7.9)	2.9 (8.6)	1.7 (7.0)	<.001^d^
Baseline neuroticism score, mean (SD)	21.1 (29.2)	25.4 (28.5)	18.7 (25.8)	<.001^d^
Personal history of depression	43.8 (39.3-48.3)	56.0 (48.3-63.8)	37.1 (31.6-42.6)	<.001^e^
Immediate family member with depression	47.1 (42.6-51.6)	52.9 (45.2-60.7)	43.9 (38.3-49.4)	.06^e^
Prior treatment for depression	32.2 (26.0-38.5)	36.5 (27.1-45.8)	28.7 (20.4-37.0)	.22^e^
Survey year^h^				
2007	11.6 (9.0-14.3)	10.8 (6.3-15.3)	12.1 (8.9-15.4)	.29^e^
2008	25.1 (21.5-28.7)	27.4 (21.3-33.5)	23.8 (19.4-28.2)	
2009	0.4 (0-1.3)	0	0.7 (0-2.0)	
2010	1.7 (0.6-2.8)	2.0 (0-4.6)	1.6 (0.5-2.6)	
2011	6.4 (3.9-8.9)	6.9 (2.5-11.2)	6.2 (3.0-9.3)	
2012	10.1 (7.3-12.9)	7.7 (3.8-11.7)	11.4 (7.6-15.1)	
2013	20.2 (16.0-24.4)	23.3 (15.8-30.8)	18.4 (13.4-23.5)	
2014	1.7 (0.8-2.6)	1.0 (0.2-1.7)	2.1 (0.7-3.5)	
2015	1.6 (0.8-2.4)	1.8 (0.3-3.3)	1.5 (0.5-2.4)	
2016	1.4 (0.4-2.4)	1.7 (0-4.1)	1.2 (0.3-2.0)	
2017	8.9 (6.4-11.3)	6.6 (3.3-9.9)	10.1 (6.8-13.4)	
2018	3.3 (2.1-4.6)	1.3 (0.4-2.3)	4.5 (2.5-6.4)	
2019	3.3 (1.7-5.0)	3.0 (0.4-5.7)	3.5 (1.4-5.6)	
2020	4.3 (2.5-6.0)	6.4 (2.5-10.3)	3.1 (1.4-4.7)	

Abbreviation: PHQ-9, 9-Item Patient Health Questionnaire.

^a^ Due to the weighting of data with truncation, only percentage estimates (95% CIs) are provided based on the weighted sample size.

^b^ *P* < .05 (2-sided) was considered significant.

^c^ Missing for 21 participants.

^d^ *t* Test with weight adjustment.

^e^ Rao-Scott χ^2^ test.

^f^ Other includes responses of American Indian or Alaska Native, Arab or Middle Eastern, Pacific Islander, other race or ethnicity, or multiple races or ethnicities on the ethnicity question of the baseline survey.

^g^ Missing for 4 participants.

^h^ *P* value computed without 2009.

### Participant Characteristics

Physicians who experienced new-onset depressive symptoms during their intern year were more likely to be female, to report higher baseline PHQ-9 and NEO scores, and to have a personal or family history of depression (Table). No association was observed between depressive symptomatology during the intern year and a prior history of receiving medication or psychotherapy for depression.

### Persistent Depression

**Figure 2.**
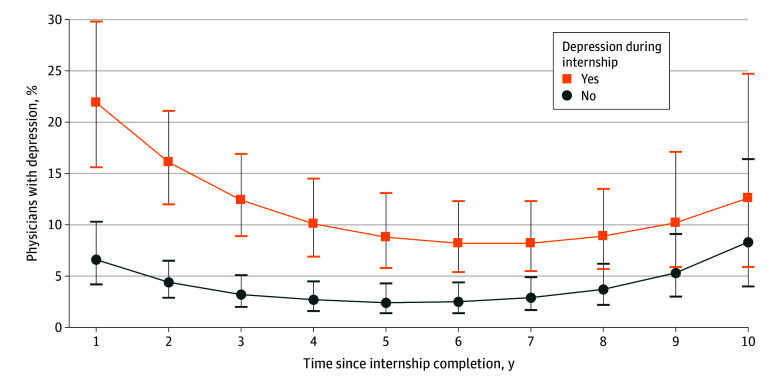
Proportion of Participants With Depression Over Time Generalized estimating equations were used, adjusted for demographic characteristics, cohort year, baseline neuroticism, and history of depression (N = 858), with weighting.

We compared the proportion of physicians with an elevated PHQ-9 score (≥10) indicating moderate to severe depressive symptoms at each year of annual follow-up between those who did and did not have an elevated PHQ-9 score during their intern year. The proportion of physicians who exceeded this threshold was higher at every year of follow-up among those who screened positive for depression at least once during their intern year compared with those who did not. A total of 21.9% of participants (95% CI, 15.6%-29.8%) who screened positive for depression during the internship also screened positive 1 year after completing their intern year. In contrast, only 6.6% (95% CI, 4.2%-10.3%) of the cohort with a PHQ-9 score of less than 10 during their intern year screened positive for depression in the first year of follow-up (Figure 2 and eTable 2 in Supplement 1). At 5 years after their internship, 8.8% of participants (95% CI, 5.8%-13.1%) who screened positive for depression during their intern year continued to exceed this PHQ-9 threshold, compared with just 2.4% (95% CI, 1.4%-4.3%) in the cohort without elevated PHQ-9 scores during their intern year (*P* < .001). Eight years after internship completion, reflecting the early years of independent practice for most participants, 8.9% of interns (95% CI, 5.7%-13.5%) with an elevated PHQ-9 score (≥10) during their intern year still exceeded this threshold, compared with just 3.7% (95% CI, 2.2%-6.2%) among those without an elevated score during their intern year (*P* = .015; Figure 2 and eTable 2 in Supplement 1).

### Mean PHQ-9 Scores and Depressive Symptoms Over Time

**Figure 3.**
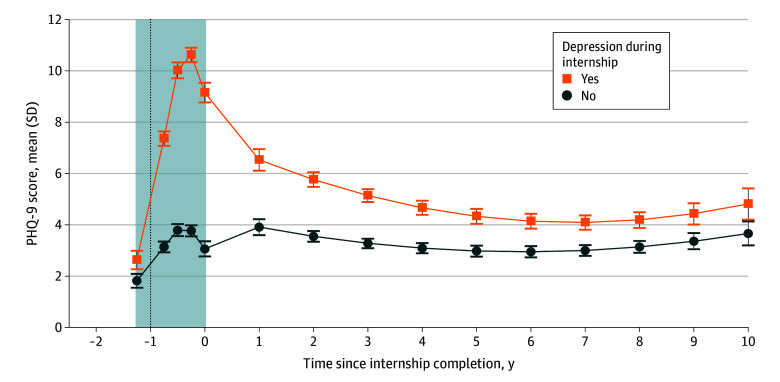
Trajectory of Mean 9-Item Patient Health Questionnaire (PHQ-9) Scores Over Time A linear mixed model was used, adjusted for demographic characteristics, baseline neuroticism, and history of depression (N = 858), with weighting. The vertical dotted line denotes the beginning of the internship year, and the shaded area indicates the quarterly survey data collected before and during the internship year.

Among physicians with new-onset depressive symptoms as interns, mean PHQ-9 scores remained higher throughout follow-up compared with those without an elevated PHQ-9 score (<10) throughout their internship. Although it did not exceed the standard PHQ-9 threshold of 10 suggestive of moderate to severe depression, the mean PHQ-9 score at 1 year after internship completion was nearly 2-fold higher in the group who had experienced more depressive symptoms as interns (6.5 [95% CI, 6.1-6.9] vs 3.9 [3.6-4.2]; *P* < .001). This difference remained statistically significant across all 10 years of follow-up (Figure 3 and eTable 3 in Supplement 1). For example, among interns who screened positive for depression (PHQ-9 score ≥10) during their internship, mean PHQ-9 scores were significantly higher at both 5 years (4.7 [95% CI, 4.4-5.0] vs 2.8 [95% CI, 2.5-3.0]; *P* < .001) and 10 years (5.1 [95% CI, 4.5-5.7] vs 3.5 [95% CI, 3.0-4.0]; *P* < .001) of follow-up. The mean PHQ-9 scores for both groups never returned to baseline (Figure 3). At 10 years after internship completion, physicians who screened positive for depression during their internship still had higher rates of positive depression screening (12.6% [95% CI, 5.9%-24.7%] vs 7.7% in the general population).

## Discussion

To our knowledge, this study is the first to explore the potential persistence of depressive symptoms after a physician’s first year in training in the US. Although previous studies have described an increase in the level of depressive symptoms during the first year of training, prior studies have not tracked these symptoms as physicians progress through training and into practice.^3^ Using a prospective cohort design and a nationally representative sample of physicians, we observed that the development of moderate to severe depressive symptoms as an intern was associated with worse long-term mental health outcomes. These findings highlight that the early years of medical training not only result in higher transient levels of depression, but they also may have lasting implications for the long-term health of the physician workforce.

Notably, screening positive for depression during the intern year was associated with a higher likelihood of screening positive for depression after completing the first years of medical training. The most significant difference between groups was noted 1 year after completion of the internship, with 21.9% (95% CI, 15.6%-29.8%) of physicians with an elevated PHQ-9 score (≥10) as interns still exceeding this threshold for depression, a level more than 3-fold higher than that for physicians who did not screen positive for depression during their internship (6.6% [95% CI, 4.2%-10.3%]; *P* < .001). Although rates of positive depression screening decreased steadily over time, physicians who screened positive for depression during their internship still had higher rates of positive depression screening 10 years after internship completion. In addition to rates higher than their physician peers, these physicians also exceeded the prevalence of depression among similarly aged adults in the general population (12.6% [95% CI, 5.9%-24.7%] in this study vs 7.7% in the general population).^16^

The second key finding relates to mean PHQ-9 scores between groups and for the entire study population. For both groups of interns, PHQ-9 scores remained higher than their baseline (before residency). However, mean scores of both groups returned to well below the elevated PHQ-9 threshold (≥10) by the completion of training. Interns with more depressive symptoms continued to have a higher burden of depressive symptoms long after completion of training. After the initial spike in depressive symptoms during the internship (Figure 3, vertical line), there was a steady decrease in PHQ-9 scores for both groups. Yet the difference in means between groups remained statistically significant throughout the follow-up period. Taken collectively, these findings suggest that depressive symptoms early in training may often persist throughout a physician’s early career trajectory.

Our findings have many important implications. These results suggest that accepting depressive symptoms among training doctors as commonplace may translate to increased depressive symptoms for nearly a decade among practicing physicians. Physician mental health was exacerbated during the recent COVID-19 pandemic, but our findings suggest that medical trainees have higher rates of depressive symptoms that have long preceded the pandemic. Finally, our findings suggest (to our knowledge, for the first time) that addressing resident mental health may improve the long-term mental health of our professional workforce.

Sleep duration and hours worked are both associated with an increased risk of depression among medical trainees. Early work from the IHS demonstrated that for each additional hour of work, the risk of depression increased by 5%; for each hour of less sleep, the risk of depression increased by 59%, even when accounting for an individual’s sleep quality measured before internship training.^17^ Although duty hours have improved in US residency programs (albeit not equally across all specialties), substantial work remains in this domain.^18^ Recent work from our group and others suggests that reducing duty hours has a protective effect on the development of depressive symptoms during training.^6,19,20,21^ Although this solution is not simple and all-encompassing, future research should examine whether depression during the intern year is prevented by reduced duty hours and increased hours slept and whether this, in turn, improves the long-term mental health of physicians.

Our research also highlights the need to better support mental health among physicians as they progress through training and to destigmatize mental health care within our professional culture. Universal well-being needs assessments for counseling services, opt-out counseling programs, and autoenrollment of interns in mental health resources to decrease barriers to access and overcome stigma should be further considered as potential solutions.^22,23^ Beyond efforts targeted at individuals, ongoing work to address workplace culture for training physicians should be widely implemented.^19,20,24^ Systems-based interventions may include further evaluation of the feedback and assessment methods and recognition of the importance of allowing time to practice basic preventative health care. Understanding these interconnected elements is imperative to understanding the relationship between residency and the mental health of residents and the workforce.

Many future areas of research are critical for reducing depression among US physicians. Further testing of the kindling hypothesis, or the concept that an initial episode of depression may serve as an independent risk factor for future depressive episodes, should be pursued using methods to support causal inferences (eg, target trial emulation, instrumental variables). Future research may also examine how mental illness affects workforce sustainability, including potential physician shortages in critical areas such as general surgery and primary care. Further research is needed to better understand the association between depression and attrition and to explore potential consequences on physician shortages. Routine screening for depression may also be considered to help identify and target interventions for those most at risk of developing depression. Finally, focused attention is necessary for subgroups underrepresented in the literature, specifically gender-diverse and nonheterosexual groups. Emerging evidence indicates that sexual minority (ie, bisexual or homosexual) individuals in medical training experience higher levels of depression than their heterosexual counterparts.^25^ Previous studies involving IHS participants reported that sexual minority individuals enter residency with higher PHQ-9 scores than their heterosexual peers and experience a more pronounced increase in depressive symptoms throughout their internship.^26^ As inclusivity in our profession increases, there is an ongoing need to support this diverse workforce appropriately.

### Limitations

This study has some limitations. The observational design limits the establishment of causal relationships. We cannot determine whether depression arising during the intern year increases a physician’s risk of later depression or whether depression that surfaces during the internship is a marker for underlying risk. Although the PHQ-9 used on both quarterly and annual surveys has high sensitivity and specificity in assessing diagnoses of major depressive disorder according to *Diagnostic and Statistical Manual of Mental Disorders, Fifth Edition*, criteria, we recognize that our interpretations are limited to the PHQ-9 outcome measure as opposed to a clinical diagnosis of depression.^15^ Selection bias may also be implicated, given that study initiation and continued participation were based on voluntary enrollment. Statistically, we aimed to minimize this bias by including weights in our analysis.

## Conclusions

The findings of this cohort study underscore that the increase in depressive symptoms observed during medical internships, although most notable in the first year of training, may persist for many trainees and physicians. This research suggests that there may be lasting consequences of depressive symptoms well beyond the years spent in medical training, emphasizing the need to support training doctors to safeguard the long-term health of those entrusted to ensure the health of others.

## Notes

Supplement 1. **eTable 1.** Annual Survey Completion by Cohort Year **eTable 2.** Percentage Screening Positive for Depression **eTable 3.** Mean 9-Item Patient Health Questionnaire (PHQ-9) Trajectory **eMethods.** SAS Code for Longitudinal Analysis of the 9-Item Patient Health Questionnaire (PHQ-9)

Supplement 2.
Data Sharing Statement
