# Supplementary material for: Duration of New-Onset Depressive Symptoms During Medical Residency
Source: JAMA Netw Open. 2024 Jun 21;7(6):e2418082. doi: 10.1001/jamanetworkopen.2024.18082 (PMC11193122; doi:10.1001/jamanetworkopen.2024.18082)
Supplement: Supplement 1. — eTable 1. Annual Survey Completion by Cohort Year eTable 2. Percentage Screening Positive for Depression eTable 3. Mean 9-Item Patient Health Questionnaire (PHQ-9) Trajectory eMethods. SAS Code for Longitudinal Analysis of the 9-Item Patient Health Questionnaire (PHQ-9) [file jamanetwopen-e2418082-s001.pdf]

Supplementary Online Content

Kim E, Sinco BR, Zhao J, et al. Duration of new-onset depressive symptoms during medical residency. *JAMA Netw Open*. 2024;7(6):e2418082. doi:10.1001/jamanetworkopen.2024.18082

eTable 1. Annual Survey Completion by Cohort Year

eTable 2. Percentage Screening Positive for Depression

eTable 3. Mean 9-Item Patient Health Questionnaire (PHQ-9) Trajectory

eMethods. SAS Code for Longitudinal Analysis of the 9-Item Patient Health Questionnaire (PHQ-9)

This supplementary material has been provided by the authors to give readers additional information about their work.

© 2024 Kim E et al. *JAMA Network Open.*

eTable 1. Annual Survey Completion by Cohort Year

Cohort Year	Number of Annual Surveys
2007	515
2008	1238
2009	2
2010	59
2011	155
2012	325
2013	158
2014	62
2015	43
2016	32
2017	143
2018	68
2019	28
2020	39
Total	2867

© 2024 Kim E et al. *JAMA Network Open.*

eTable 2. Percentage Screening Positive for Depression

Generalized Estimating Equation, Percent (95% CI), N= 858, Data Through 2022, Adjustment for Demographics, Cohort Year, Baseline Neuroticism, and History of Depression, With Weighting

Time Since Completion of Internship (Years)	Positive Depression Screen (PHQ>/=10) During Intern Year (N = 302)	No Positive Depression Screen During Intern Year. (N = 556)	Entire Cohort N = 858	P-Value[1]
1	21.9 (15.6, 29.8)	6.6 (4.2, 10.3)	11.9 (9.1, 15.3)	<.001
2	16.1 (12.0, 21.1)	4.4 (2.9, 6.5)	8.4 (6.6, 10.5)	<.001
3	12.4 (8.9, 16.9)	3.2 (2.0, 5.1)	6.3 (4.9, 8.2)	<.001
4	10.1 (6.9, 14.5)	2.7 (1.6, 4.5)	5.2 (3.8, 7.0)	<.001
5	8.8 (5.8, 13.1)	2.4 (1.4, 4.3)	4.6 (3.3, 6.4)	<.001
6	8.2 (5.4, 12.3)	2.5 (1.4, 4.4)	4.5 (3.2, 6.2)	0.001
7	8.2 (5.5, 12.3)	2.9 (1.7, 4.9)	4.7 (3.4, 6.5)	0.003
8	8.9 (5.7, 13.5)	3.7 (2.2, 6.2)	5.4 (4.0, 7.4)	0.015
9	10.2 (5.9, 17.1)	5.3 (3.0, 9.1)	6.8 (4.7, 9.7)	0.120
10	12.6 (5.9, 24.7)	8.3 (4.0, 16.4)	9.2 (5.6, 14.7)	0.456

[1]A p-value of less than .05 was considered significant, and all p-values were 2-sided.

© 2024 Kim E et al. *JAMA Network Open.*

eTable 3. Mean 9-Item Patient Health Questionnaire (PHQ-9) Trajectory

Linear Mixed Model, Mean (95% CI), Adjusted for Demographics, Cohort Year, Baseline Neuroticism, History of Depression, N= 858, With Weighting

**Time Since Completion of Internship (Years)**	**Positive Depression Screen Episode During Intern Year (N = 302)**	**No Positive Depression Screen During Intern Year. (N = 556)**	**Entire Cohort N = 858**	**P-Value[1]**
Pre-Internship Interview	2.6 (2.3, 3.0)	1.8 (1.5, 2.1)	2.1 (1.8, 2.3)	<.001
-1	7.4 (7.1, 7.6)	3.1 (2.9, 3.4)	4.6 (4.4, 4.9)	<.001
-0.75	10.0 (9.7, 10.3)	3.8 (3.6, 4.0)	6.1 (5.8, 6.3)	<.001
-0.5	10.6 (10.3, 10.9)	3.8 (3.6, 4.0)	6.3 (6.1, 6.5)	<.001
0	9.2 (8.8, 9.5)	3.1 (2.8, 3.4)	5.4 (5.1, 5.6)	<.001
1	6.5 (6.1, 6.9)	3.9 (3.6, 4.2)	4.9 (4.6, 5.1)	<.001
2	5.8 (5.5, 6.0)	3.6 (3.3, 3.8)	4.4 (4.2, 4.6)	<.001
3	5.1 (4.9, 5.4)	3.3 (3.1, 3.5)	4.0 (3.8, 4.1)	<.001
4	4.7 (4.4, 4.9)	3.1 (2.9, 3.3)	3.7 (3.5, 3.8)	<.001
5	4.3 (4.0, 4.6)	3.0 (2.8, 3.2)	3.5 (3.3, 3.7)	<.001
6	4.1 (3.8, 4.4)	2.9 (2.7, 3.2)	3.4 (3.2, 3.6)	<.001
7	4.1 (3.8, 4.4)	3.0 (2.8, 3.2)	3.4 (3.2, 3.6)	<.001
8	4.2 (3.9, 4.5)	3.1 (2.9, 3.4)	3.5 (3.3, 3.7)	<.001
9	4.4 (4.0, 4.8)	3.4 (3.0, 3.7)	3.7 (3.5, 4.0)	<.001
10	4.8 (4.2, 5.4)	3.7 (3.2, 4.1)	4.1 (3.6, 4.5)	0.003

[1]A p-value of less than .05 was considered significant, and all p-values were 2-sided.

© 2024 Kim E et al. *JAMA Network Open.*

eMethods. SAS Code for Longitudinal Analysis of the 9-Item Patient Health Questionnaire (PHQ-9)

/* Interrupted Time Series, Adjust for Demographics, Baseline Neuroticism, History of Depression, Cohort Year */

```
/* Explanation of Variables */
/* Age = age in years */
/* Depr0Bin = 1 if history of depression, 0 if not */
/* EthBlack = 1 if African American / Black, 0 if not */
/* EthHispLat = 1 if Hispanic/LatinX, 0 if not */
/* EthAsian = 1 if Asian, 0 if not */
/* EthOtherUnk = 1 if other or unknown race/ethnicity */
/* Female = 1 if female, 0 if male */
/*  InternDepress_YN =1 if depressive episode during intern year, 0 if not */
/* Neu0 = baseline neuroticism score */
/* PHQtot = total PHQ-9 score */
/* TimeInYears = time in years from baseline interview */
/* TYMinus1 = TimeInYears - 1 */
/* TYMinus1sq = (TimeInYears - 1)^2 */
/* YearLE1 = 1 if TimeInYears ≤ 1, 0 otherwise */
/* TimeInYears2 = TimeInYears^2 */
/*  YearGT1 = 1 if TimeInYears > 1, 0 otherwise */
/* UserID = participant ID number */
/* Weight_Final483 = final weight, truncated at 95th percentile */
/* Year = cohort year */

ods graphics on;
Proc Mixed Data=PHQLong Method=REML noclprint plots=all;
  Class UserID Year;
  Weight Weight_Final483;
  Model PHQtot = Age Female EthBlack EthHispLat EthAsian EthOtherUnk
  InternDepress_YN TimeInYears*YearLE1 InternDepress_YN*TimeInYears*YearLE1
  TimeInYears2*YearLE1 InternDepress_YN*TimeInYears2*YearLE1
  YearGT1 InternDepress_YN*YearGT1 TYMinus1*YearGT1 InternDepress_YN*TYMinus1*YearGT1
  TYMinus1sq*YearGT1 InternDepress_YN*TYMinus1sq*YearGT1
  Neu0 Depr0Bin Year/ solution ddfm=kr2;
Repeated / Type=Sp(Pow)(TimeInYears) subject=UserID;
Where TimeInYears<=11;
Run;
```

SAS Code for Longitudinal Analysis of the binary variable for (PHQ-9 ≥ 10)

```
/* Explanation of Variables */
/* PHQGE10 = 1 if PHQ-9 score ≥ 10, 0 otherwise */
/* TYMinus2 = (TimeInYears - 2), 2 years from baseline is 1 year after completion of internship*/
/* TYMinus2sq = (TimeInYears - 2)^2 */

ods graphics on;
Proc GenMod Data=PHQLong Descending plots=all;
Class UserID Year;
Weight Weight_Final483;
Model PHQGE10 =  Age Female EthWhite
```

© 2024 Kim E et al. *JAMA Network Open.*

```
  InternDepress_YN TYMinus2 TYMinus2sq InternDepress_YN*TYMinus2
InternDepress_YN*TYMinus2sq  Neu0 Depr0Bin Year/ dist=bin;
Repeated subject=UserID / Type=CS;
Where TYMinus2>=0 and TimeInYears<=11; /* 1 to 10 years after completion of internship */
Run;
```

© 2024 Kim E et al. *JAMA Network Open.*
