# Supplementary material for: Duration of New-Onset Depressive Symptoms During Medical Residency
Source: JAMA Netw Open. 2024 Jun 21;7(6):e2418082. doi: 10.1001/jamanetworkopen.2024.18082 (PMC11193122; doi:10.1001/jamanetworkopen.2024.18082)
Supplement: Supplement 2. — Data Sharing Statement [file jamanetwopen-e2418082-s002.pdf]

# Data Sharing Statement

Kim. Duration of New-Onset Depressive Symptoms During Medical Residency. *JAMA Netw Open*. Published June 21, 2024. doi:10.1001/jamanetworkopen.2024.18082

**Data**
**Data available:** Yes
**Data types:** Deidentified participant data
**How to access data:** https://www.openicpsr.org/openicpsr/project/129225/version/V1/view
**When available:** beginning date: 01-12-2024

**Supporting Documents**
**Document types:** None

**Additional Information**
**Who can access the data:** anyone who requests may access data
**Types of analyses:** any purpose
**Mechanisms of data availability:** Publically available on website
